# Case report of synchronous post-lung transplant colon cancers in the era of colorectal cancer screening recommendations in cystic fibrosis: screening “too early” before it’s too late

**DOI:** 10.1186/s12876-019-1052-7

**Published:** 2019-07-29

**Authors:** James M. Abraham, Kathleen Mahan, Tetyana Mettler, Jordan M. Dunitz, Alexander Khoruts

**Affiliations:** 1 Department of Medicine, Division of Gastroenterology, University of Minnesota, Minneapolis, MN USA; 2 Department of Medicine, Division of Pulmonary Medicine, University of Minnesota, Minneapolis, MN USA; 3 Department of Pathology, University of Minnesota, Minneapolis, MN USA

**Keywords:** Cystic fibrosis (CF), Colorectal cancer (CRC), Lung transplantation, Screening, Surveillance, Guidelines

## Abstract

**Background:**

*The increasing life expectancy of individuals with Cystic Fibrosis (*CF*) is likely to be associated with new age-related challenges, colorectal cancer (CRC) most notably; recent consensus recommendations for CRC screening published in 2018 represent an important early step in addressing the emerging awareness of* CF *as a gastrointestinal cancer syndrome. These recommendations, however, need to be further refined based on more systematic data. We discuss an illustrative first-ever case of synchronous CRC arising in a post-lung transplant individual with* CF *within the recommended surveillance interval after a well-documented prior normal colonoscopy.*

**Case presentation:**

*A 51-year-old female individual with homozygous F508del* CF*, presents to clinic with abdominal discomfort and intermittent blood in stools. She had previously undergone bilateral lung transplantation 18 years earlier, as well as two kidney transplants related to immunosuppression-related nephrotoxicity. A diagnostic colonoscopy was performed which revealed the presence of two separate synchronous colon cancers in the cecum and transverse colon; she had undergone a colonoscopy three years prior to this exam which was structurally normal. Endoscopic quality indicators, including a good quality bowel preparation, colonoscopic withdrawal time > 12 min, and quarterly Adenoma Detection Rate (ADR) ranging from 50 to 70% for both male and female patients for the endoscopist from both colonoscopic exams, as well as secondary retrospective comparative review of the pertinent case images, diminish the risk for a “missed” cancer or advanced lesion on the index exam. These cancers did not demonstrate any immunohistochemical features suggestive of Lynch Syndrome, though the rapid progression to cancer within the surveillance interval (possibly non-polypoid in nature) is similar. This cancer presentation within the newly-established recommended colon cancer screening interval warrants concern.*

**Conclusions:**

*This case prompts serious discussion regarding the length of surveillance intervals in the post-transplant* CF *population (a population at 20–30 times greater risk for CRC compared to the general non-*CF *population), as well as the importance of documenting endoscopic quality benchmarks, particularly if a narrative of interval CRC development continues to develop with further prospective monitoring and multi-center experience.*

## Background

The increasing life expectancy of individuals with Cystic Fibrosis (CF) is likely to be associated with new age-related challenges, colorectal cancer (CRC) most notably; recent consensus recommendations for CRC screening published in 2018 represent an important early step in addressing the emerging awareness of CF as a gastrointestinal cancer syndrome. These recommendations, however, need to be further refined based on more systematic data. We discuss an illustrative first-ever case of synchronous CRC arising in a post-lung transplant individual with CF within the recommended surveillance interval after a well-documented prior normal colonoscopy.

## Case presentation

A 51-year-old female individual with Cystic Fibrosis (CF), homozygous F508del, presented to the Minnesota Cystic Fibrosis Center Gastroenterology Clinic with persistent abdominal bloating and loose stools worsening over several months. The patient underwent bilateral lung transplantation almost 20 years prior to clinic presentation for progressive pulmonary function decline related to CF. Her post-transplant course was complicated by chronic kidney disease progressing to renal failure associated with calcineurin inhibitor use for which she underwent two living donor kidney transplantations, six years and ten years after lung transplantation. Her immunosuppressive regimen on presentation included tacrolimus, mycophenolate mofetil, and prednisone, and she had excellent ongoing lung and renal graft function. The patient also had CF-related diabetes (CFRD) and exocrine pancreatic insufficiency (EPI), both well-managed with subcutaneous insulin injections and pancreatic enzyme replacement therapy (PERT).

The patient was evaluated for similar symptoms three years prior to the current presentation. Diagnostic evaluation at that time included a negative upper endoscopy and ileocolonoscopy; a bowel preparation adequate for lesion detection was achieved utilizing a “multiple-wash” aggressive approach per the University of Minnesota CF protocol [1]. Family history was negative for gastrointestinal malignancies and associated syndromes. The patient had not undergone any prior screening or diagnostic endoscopic evaluations. Following this evaluation, with a slight increase in her PERT supplementation (though total PERT dosage remained well less than 10,000 units of lipase/kg/day), and in concerted effort with a CF nutritionist for oral nutritional supplementation, the patient progressively improved with a plan for as-needed follow-up in the Gastroenterology Clinic.

At her current presentation, a detailed History & Physical Exam was performed which was otherwise unchanged compared to her clinic visit three years prior, with the exception of the patient reporting the presence of small amounts of bright red blood intermittently with passage of some stools. A colonoscopy was recommended in view of the new symptom (overt rectal bleeding) and her post-transplant immunosuppressed state, which has been associated with a greater risk of colorectal cancer [2].

**Fig. 1:**
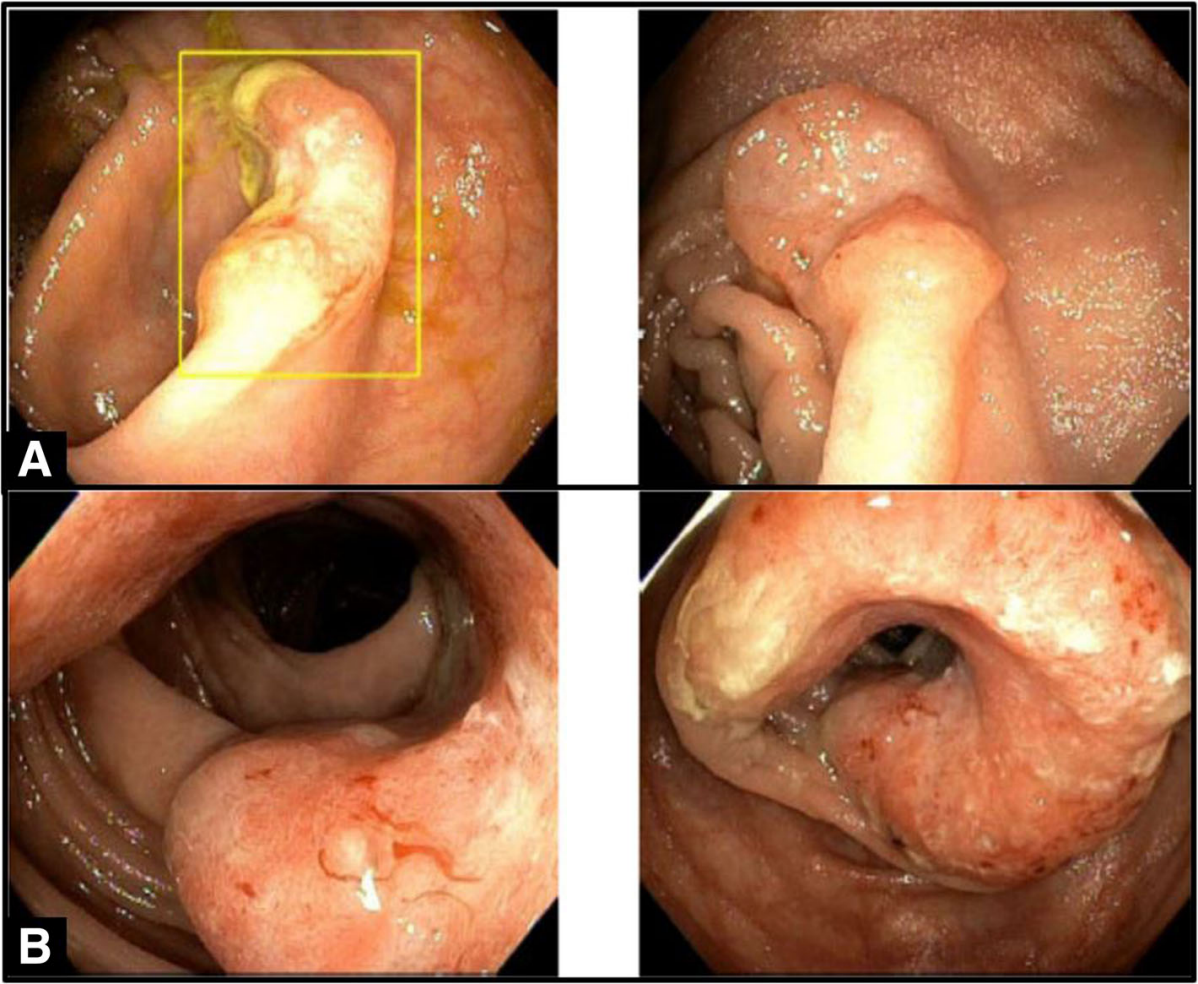
Endoscopic. Picture A (top): Cecal adenocarcinoma (in box), characterized by an invasively-ulcerated discrete nodule. Picture B (bottom): Distal transverse colon adenocarcinoma, characterized by near-circumferential non-obstructing protrusion several centimeters in length

The colonoscopy revealed two separate masses located in the cecum and distal transverse colon. The cecal mass, characterized as an ulcerated invasive nodule, was 1.2 cm in size; the transverse colon mass was partially circumferential, fungating, friable, and measured 5 cm in length. *(*Fig. 1*)* There was reasonable concern raised whether the transverse colon lesion could have represented a “missed” interval cancer despite the normal prior exam (false-negative result). Images from the first exam performed three years prior (photo-documented through the terminal ileum, cecum, right colon, transverse colon, left colon, and rectum) were compared to images from the subsequent exam. No obvious lesions or mucosal abnormalities were noted in the transverse colon or other colonic segments in retrospect (though this was not definitively excluded), and the cecal lesion was definitively confirmed by photo comparison to be a new lesion not previously present in either adenomatous form or mucosal abnormality on the prior exam. Quarterly adenoma detection rates (ADR) for the endoscopists for the periods when each exam occurred ranged between 50 and 70% for both male and female patients (standard-of-practice acceptable ADR is at least > 25% for both male and female patients). A multi-day/multi-wash split bowel preparation was utilized per the University of Minnesota CF Bowel Preparation Protocol, and was documented in the procedure reports (and confirmed on image review) as of “good quality” to identify polyps ≥5 mm in size. Colonoscopic withdrawal inspection time was > 12 min for both exams (standard-of-practice acceptable withdrawal inspection time is at least > 6 min). While the risk of a false-negative result with any screening or surveillance regimen is not zero, confirmation of adequacy of these endoscopic benchmarks would typically indicate a diminished risk for this possibility [3–5].

**Fig. 2:**
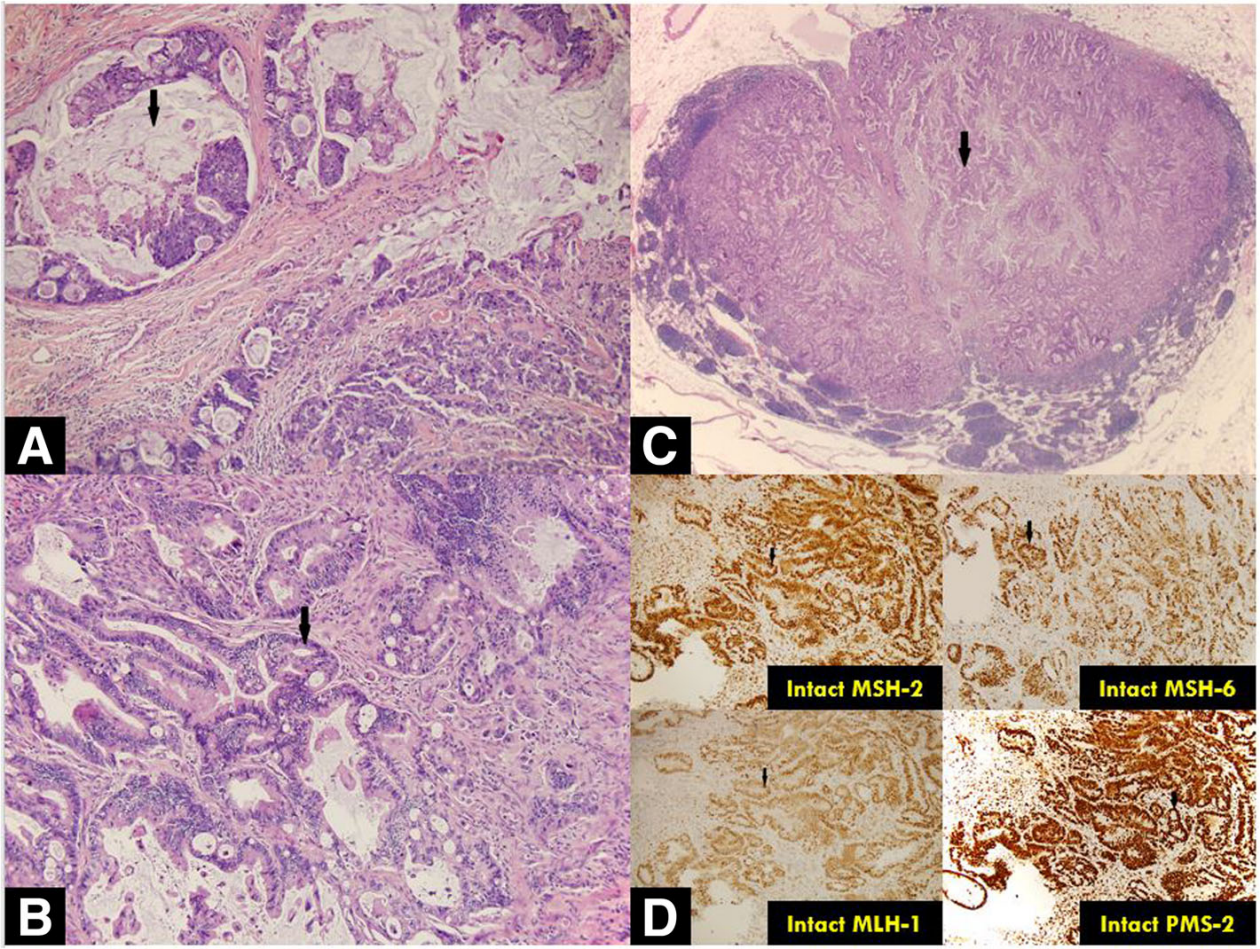
Histologic. Picture A: Cecal adenocarcinoma displaying mucinous features, mucin (indicated by arrow). Picture B: Distal transverse colon adenocarcinoma, infiltrating malignant glands (indicated by arrow). Picture C: Metastatic adenocarcinoma involving regional lymph node, metastatic adenocarcinoma glands (indicated by arrow). Picture D: Intact mismatch repair proteins by immunohistochemical tumor staining, Lynch Syndrome unlikely

Biopsies of each of the masses confirmed synchronous, separate, invasive, moderately-to-poorly differentiated (cecal mass) and moderately differentiated (transverse colon mass) adenocarcinomas. Staging computed tomography (CT) scan did not reveal overt evidence of metastases and a referral was made to the Oncology and Colorectal Surgery services with anticipation of treatment with curative intent. Subtotal colectomy with splenic flexure mobilization and ileal descending anastomosis was subsequently performed. The cecal nodule demonstrated moderate-to-poorly differentiated adenocarcinoma with submucosal invasion. The transverse colon lesion revealed visceral peritoneal and lymphovascular invasion with clean resection margins; three of 60 lymph nodes returned positive for tumor involvement. Mismatch repair protein expression was normal in both lesions. *(*Fig. 2*)* Initial post-operative recovery was relatively uneventful, and the patient was discharged home on postoperative day (POD) seven. She was readmitted, however, on POD 11 with hypoxic respiratory failure and septic shock of unclear etiology; lung (bilateral infiltrates on chest radiograph) and intra-abdominal sources (peritoneal free air following recent surgery) were suspected. After a complicated hospitalization, she was ultimately transitioned to comfort cares and passed away with her family at her bedside.

This may be the first case of post-transplant CRC in a long-term multi-transplant survivor reported ***within the proposed surveillance interval after a normal colonoscopy*** following publication of the recent CF CRC screening consensus recommendations from the Cystic Fibrosis Foundation (Bethesda, MD, USA) [6]. The authors feel that this case may contribute to the ongoing multi-national discussions and prospective monitoring of outcomes under the currently recommended CRC screening and surveillance intervals in individuals with CF, particularly in those individuals with CF who have undergone prior lung (or other solid organ) transplantation.

## Discussion and conclusions

Recently CF has become more distinctly recognized as an inherited colorectal cancer (CRC) syndrome [2, 7–10]. The risk of CRC in individuals with CF appears to be 5–10 times higher relative to the general population, and even greater in individuals with CF on immunosuppression following organ transplantation (an almost 30-fold increased risk relative to the general population) [2, 10–12]. The recently published CRC Screening Consensus Recommendations by the United States (US) Cystic Fibrosis Foundation (CFF) endorse colonoscopy as the preferred screening modality in individuals with CF, starting at age 40 in absence of an organ transplant (Table 1). Organ transplant recipients are recommended to start CRC screening at an earlier age with similar interval screening guideline recommendations. The recommendations emphasize individualization of screening and surveillance intervals based on multiple patient-specific factors, including co-morbidities, quality of colonoscopic preparation, and specific characteristics/nature of polyps identified. Furthermore, the recommendations underscore that new gastrointestinal symptoms should prompt consideration of bowel malignancy and earlier endoscopic evaluation.

**Table 1:** 2018 CFF Colorectal Cancer Screening recommendations in Cystic Fibrosis

CFF CRC Screening REcommendations (colonoscopy advised)	INTERVAL	Additional Information
40 yrs		Individuals with CF
30 yrs		Individuals with CF and organ transplant, at least 2 years after transplant if no screening in the prior 5 years
Negative colonoscopy	Every 5 years	
Adenomatous polyp	Every 3 years	
All colonoscopies		Intensive bowel preparation including 3–4 washes (1 L purgative per wash) and last wash within 4–6 h of exam

The currently recommended screening and surveillance intervals for colonoscopic examinations in individuals with CF are somewhat shorter relative to the general population, but longer relative to most other hereditary CRC syndromes [13]. The development of CF CRC screening consensus recommendations had to consider various factors, including the many co-morbidities associated with CF, the shortened life expectancy, the burden of colonoscopic examinations that require rigorous preparations, and the relative paucity of systematic data on the outcomes of screening examinations to date. Earlier development and increased incidence of adenomatous polyps in adults with CF has been reported by different centers, including those in the US, Australia, and Canada [1, 14–17]. Our own center formalized its CRC screening protocol in 2010, at which time all patients began to receive uniform recommendations for screening and surveillance, including the rigorous CF-specific colonoscopic preparation instructions. The accumulated single center experience demonstrated that CF is associated with earlier formation of colon adenomas and their faster progression to advanced lesions as defined by size and histopathology [18]. Notably, advanced polyps (defined as size ≥1 cm or presence of villous histopathology, high-grade dysplasia, or carcinoma in-situ) could be found in ~ 25% of patients on surveillance colonoscopies done at 1–2 year intervals.

Current recommendations stratify transplant and non-transplant patients with respect to the timing of initiation of screening. However, the screening and surveillance intervals are identical. It is notable that the relative risk of CRC in transplanted individuals with CF is greater than that in the Lynch syndrome, the most common hereditary CRC syndrome [17]. The interval for colonoscopic surveillance in Lynch syndrome in the current guidelines is 1–2 years [13]. This was established following demonstration of less CRC, earlier stage of CRC at diagnosis, and less CRC-related mortality with more frequent (≤ 2 years) colonoscopic examinations as compared to less frequent examinations [19–22]. The current CF CRC screening consensus recommendations are largely supported by the cost-effectiveness analysis using the microsimulation screening analysis-colon model for CRC [23]. However, the modeling determined that the optimal interval for colonoscopic re-screening for transplanted individuals with CF with negative prior examinations was 3 years between ages 35 and 55, which is shorter than the current consensus recommendations for this patient subgroup.

It is important to emphasize that the current consensus recommendations and the modeling analyses are based on very limited data. One of the biggest unknowns is the “adenoma dwell time” in individuals with CF with and without immunosuppression. Our data thus far [18], poignantly illustrated by this case, suggests markedly accelerated progression of adenomas in transplanted individuals with CF. It is noteworthy that the modeling analyses also found an annual fecal immunohistochemical test (FIT) to be a comparably cost-effective testing strategy in CF, assuming that the performance of FIT is not hindered by the underlying disease [23]; it should be noted that the operating characteristics of stool-based testing for CRC screening in a CF population are currently unknown. The impact of transplant survivorship duration, type/depth/combination of immunosuppressive exposures, and multiple-transplant status is also not well-quantified at this time in the CF population. It is possible that a hybrid strategy combining annual FIT and regular colonoscopic examinations even with negative FIT results may be the most effective means of mitigating the risk of interval CRC-associated morbidity and mortality.

The increasing life expectancy of individuals with CF is likely to be associated with new age-related challenges, CRC most notably. The current consensus recommendations for CRC screening represent an important early step in addressing the emerging awareness of CF as a gastrointestinal cancer syndrome [6]. These recommendations and screening intervals, however, if corroborated by further prospective systematic (and preferably multi-center) data, may need to be revisited. Historically, the CF patient registries have not focused on granular CRC-related information, and going forward it will be important to clearly capture data on CRC screening, incidence, and related mortality in all CF centers. Recording of the best available endoscopic quality measures of those endoscopists performing screening colonoscopy in individuals with CF will also be vital to establishing an accurate narrative of interval cancer risk in this population. These would include quarterly documentation of ADR and cecal intubation rate, as well as procedural documentation of bowel preparation adequacy and withdrawal inspection time [3–5]. This should be done while noting the inherent limitations of these measures, particularly by monitoring the incidence of interval cancers despite adequacy of these benchmarks [24].

Additionally, it will be critically important to evaluate these surveillance data in the era of cystic fibrosis transmembrane conductance regulator (CFTR) modulator therapy, particularly to determine impacts of CFTR modulation on CRC incidence based on condition penetrance, age of clinical disease onset/recognition, and treatment exposure. Regardless of the granular detail collected in cases of CRC occurring in individuals with CF, the risks of reducing the screening interval (and thereby increasing exposure to the attendant risks of colonoscopy) will first need to be carefully weighed against the existing medical burden of CF before any such change to the current formal recommendations can be made, preferably with further prospective data analysis to guide the way.

The remarkable gains in lifespan for individuals with CF continue to be a testament to the resilience of patients and their caregivers, and the commitment of their partnering multidisciplinary care teams; the advent of CFTR modulation offers further hope for additional gains to come. It will be incumbent on all involved stakeholders to proactively approach the challenges of age, and cancer prevention especially, as they arise.

## Data Availability

Not applicable.

## Abbreviations

ADR: Adenoma Detection Rate
CF: Cystic Fibrosis
CFF: Cystic Fibrosis Foundation
CFRD: Cystic Fibrosis-related Diabetes
CFTR: Cystic Fibrosis Transmembrane Conductance Regulator
CRC: Colorectal Cancer
CT: Computed Tomography
EPI: Exocrine Pancreatic Insufficiency
FIT: Fecal Immunohistochemical Test
PERT: Pancreatic Enzyme Replacement Therapy
POD: Postoperative Day
